# Special Issue: Chronic Pulmonary Aspergillosis

**DOI:** 10.3390/jof8070714

**Published:** 2022-07-08

**Authors:** Chris Kosmidis

**Affiliations:** Division of Evolution, Infection and Genomics, Faculty of Biology, Medicine and Health, Manchester Academic Health Science Centre, University of Manchester, Manchester M23 9LT, UK; chris.kosmidis@manchester.ac.uk

I would like to thank all authors who contributed to this *Journal of Fungi* Special Issue on Chronic Pulmonary Aspergillosis (CPA). CPA is becoming recognised as an important cause of morbidity in both low- and high-resource settings, and it was great to see papers from India, Indonesia, Pakistan, South Korea, Thailand, Uganda, the UK and the USA. We received both original research and reviews addressing the pathogenesis of CPA, diagnostic aspects, epidemiology and treatment.

Improving the diagnosis of CPA is the most pressing issue. In TB high-burden countries, CPA can complicate even successfully treated TB; Rozaliyani et al. [1,2] suggest that up to one in five patients with negative TB investigations following the completion of TB treatment may have CPA. A point-of-care test based on detection of Aspergillus IgG/IgM antibody (LDBio Aspergillus ICT) had 80% sensitivity and 70% specificity. This lateral flow assay has the potential to greatly enhance the diagnosis of CPA in resource-constrained countries, where laboratory-based serological tests may not be available.

In Pakistan and other Asian countries, *Aspergillus flavus* may account for the majority of CPA cases. The importance of CPA caused by this species is poorly understood. In a pilot study, Jabeen et al. evaluated for the first time the performance of *A. flavus*-specific IgG using the Siemens Immulite assay and recommended a cut off of 30 mg/L [3]. More work should follow to better characterise non-fumigatus CPA. 

In addition to TB, CPA complicates a large number of chronic lung conditions. Greater awareness is needed among clinicians in order to suspect and recognise the disease among different patient populations. For this reason, the paper by Shin et al. is a welcome addition as they highlight the risk factors to subsequently develop CPA for patients treated for lung cancer with curative intent. The cumulative incidence of CPA following surgery for lung cancer was 1.6% at five years and 3.5% at 10 years. Factors linked to CPA in cancer patients were smoking, low BMI, thoracotomy vs. VATS, interstitial lung disease and receipt of both chemotherapy and radiotherapy [4]. 

Vitamin D deficiency has been implicated as a poor prognostic factor in certain infections. Sehgal et al. did not find an association between Vitamin D deficiency and the severity of CPA, nor was Vitamin D deficiency more common in CPA vs. controls [5]. 

As CPA is a relatively rare and under-recognised disease in high-resource settings, multinational collaboration is crucial. For this reason, I welcomed the contribution by CPAnet, an international multicentre collaborative group that aims to improve CPA knowledge and patient care through research. CPAnet have established a Registry of CPA cases with input from several European countries that contributes to the better understanding of the disease [6].

Finally, it was also great to see several reviews from experts in the field: reviews on the diagnosis and management of CPA in high- and low-resource settings [7,8], on molecular diagnostic methods in CPA [9] and on the interaction of non-tuberculous mycobacterial lung disease with CPA [10].

I am delighted to present this Special Issue on CPA and I am confident it will raise awareness for this neglected disease.
